# Disrupted Service Delivery? The Impact of Conflict on Antenatal Care Quality in Kenya

**DOI:** 10.3389/fgwh.2021.599731

**Published:** 2021-02-26

**Authors:** Adanna Chukwuma, Kerry L. M. Wong, Uche Eseosa Ekhator-Mobayode

**Affiliations:** ^1^World Bank Group, Washington, DC, United States; ^2^Faculty of Epidemiology and Population Health, London School of Hygiene and Tropical Medicine, London, United Kingdom

**Keywords:** antenatal care, conflict, violence, quality of care, maternal health, Kenya

## Abstract

**Introduction:** African countries facing conflict have higher levels of maternal mortality. Understanding the gaps in the utilization of high-quality maternal health care is essential to improving maternal survival in these states. Few studies have estimated the impact of conflict on the quality of health care. In this study, we estimated the impact of conflict on the quality of health care in Kenya, a country with multiple overlapping conflicts and significant disparities in maternal survival.

**Materials and Methods:** We drew on data on the observed quality of 553 antenatal care (ANC) visits between January and April 2010. Process quality was measured as the percentage of elements of client–provider interactions performed in these visits. For structural quality, we measured the percentage of required components of equipment and infrastructure and the management and supervision in the facility on the day of the visit. We spatially linked the analytical sample to conflict events from January to April 2010. We modeled the quality of ANC as a function of exposure to conflict using spatial difference-in-difference models.

**Results:** ANC visits that occurred in facilities within 10,000 m of any conflict event in a high-conflict month received 18–21 percentage points fewer components of process quality on average and had a mean management and supervision score that was 12.8–13.5 percentage points higher. There was no significant difference in the mean equipment and infrastructure score at the 5% level. The positive impact of conflict exposure on the quality of management and supervision was driven by rural facilities. The quality of management and supervision and equipment and infrastructure did not modify the impact of conflict on process quality.

**Discussion:** Our study demonstrates the importance of designing maternal health policy based on the context-specific evidence on the mechanisms through which conflict affects health care. In Kenya, deterioration of equipment and infrastructure does not appear to be the main mechanism through which conflict has affected ANC quality. Further research should focus on better understanding the determinants of the gaps in process quality in conflict-affected settings, including provider motivation, competence, and incentives.

## Introduction

In September 2000, the United Nations Millennium Summit concluded with the adoption of the Millennium Development Goals, including a commitment to reduce the global maternal mortality ratio (MMR) by three-quarters between 1990 and 2015. Between 2000 and 2017, the global MMR fell by 38%, from 342 to 211 deaths per 100,000 live births. This progress was significantly short of the target (1). Declines in maternal mortality were also uneven between the two regions with the highest burden of disease. While the MMR in South Asia declined by 58.7% between 2000 and 2017, Sub-Saharan Africa (SSA) saw a more modest decline of 38.8% (1). About 66% of global maternal deaths still occur in SSA. Relatively higher average levels of maternal mortality have persisted in African countries facing conflict and post-conflict (1). In a 2007 study, the median-adjusted MMR was 45% higher in countries with recent conflict (2). Countries with internal conflict have also experienced greater subnational disparities in maternal outcomes (1). Therefore, understanding the determinants of maternal mortality in conflict-affected countries in SSA is essential to reducing disparities in maternal survival both across and within countries.

Good quality maternal health care is essential to maternal survival but might be particularly challenging to provide and deliver in conflict-affected contexts (1). In most cases, maternal deaths are preventable (3). An estimated 40% of pregnant women will experience obstetric disorders that are not immediately fatal given medical intervention (3, 4). However, in countries with high mortality rates, maternal interventions are often not accessible or are of suboptimal quality, leading to lost opportunities to improve outcomes. Global coverage of antenatal care (ANC) and skilled birth attendance (SBA) is above 75% on average. However, coverage in several conflict-affected states is below this average. ANC coverage falls to 9.4% in Somalia, and SBA coverage in Afghanistan is 17.8% (5). Quality-adjusted coverage is even lower in high-mortality countries. In a cross-sectional study of eight countries, the quality-adjusted coverage of ANC was 28.3%, far below the proportion of women who received any ANC at 95.7% (6). The quality of maternal health care has also been shown to predict utilization in African countries, illustrating the interconnectedness of the challenges of underutilization and poor quality (7). Health system investments that have increased maternal health care use without improving the quality of care have contributed minimally to reducing mortality. Illustratively, India's Janani Suraskha Yojana, a program aimed at removing financial barriers to giving birth in health facilities, led to a 5.6 percentage points increase in SBA in districts with higher program coverage (8). Nonetheless, there was no significant reduction in mortality in districts with higher program coverage, attributed in part to the lack of access to high-quality obstetric care (9, 10).

A clear conceptualization of quality is essential to examining its implications for health service delivery in conflict-affected contexts. Maternal health care is of high quality to the degree that these health services increase the likelihood of the desired health outcomes and are consistent with current professional knowledge (11). In a seminal publication, Donabedian described three measurable components of the quality of care (12). Structural quality refers to health care inputs, including the adequacy of facilities and equipment, the qualifications of medical staff, and management procedures. Process quality refers to the extent to which client–provider interactions are consistent with current professional knowledge, including the use of evidence-based guidelines, minimization of errors, and avoidance of delays (13). Structural and process quality interacts, as improvements in client–provider interactions correlate with the adequacy of health care inputs, including the availability of providers, and facility management. However, process quality also depends on provider competence, intrinsic motivation, and external incentives (14, 15). Outcome quality reflects the combined impact of structural and process quality on health outcomes and is the ultimate validator of high quality. There may be a time lag between the delivery of services with adequate structural and process quality and improvements in health outcomes. Outcome measures attributable to improvements in quality may also be more difficult to measure than structural and process measures, particularly in data-poor environments. Furthermore, despite adequate structural and process quality, health outcomes may not improve due to other factors beyond the control of the health facility and provider, including access to nutrition, water, and sanitation. While there are other conceptualizations of the quality of health care, we draw on Donabedian's framework in reviewing the empirical literature (13, 16).

Empirical studies of the potential impact of conflict on maternal mortality have examined its implications for health care utilization and structural and process quality. To this end, there is a significant body of research that links maternal health care use to conflict, drawing on qualitative and quantitative approaches. In a study of the Syrian conflict, DeJong et al. (17) demonstrate that ANC use fell from 87.7 to 62%, whereas SBA coverage decreased from 96.2 to 72%, following conflict exposure. Akseer et al. (18) published a time-series ecological study in 2020 that showed that inequalities in maternal health care use were higher in conflict than in non-conflict countries, with lower coverage rates among the poorest, least educated, and rural-dwelling households. Chukwuma and Ekhator-Mobayode also show that exposure to the Boko Haram insurgency reduced the probability of receiving any ANC and SBA, drawing on spatial difference-in-difference (DID) models (19). Price and Bohara document a negative correlation between ANC coverage and violent events in Nepal during the Maoist insurgency after controlling for other determinants of health care access. Reductions in ANC, SBA, and other maternal health care have also been demonstrated in Lebanon, Eastern Burma, and Uganda (20–22). Hence, overall, the empirical evidence indicates that exposure to conflict predicts falls in maternal health care utilization.

Several descriptive studies have highlighted the important implications of conflict for the supply and adequacy of facilities, equipment, and health workers, which have direct negative implications for the structural quality of health care. Studies of conflict in Nicaragua, Burundi, Northern Uganda, and Syria have described the deterioration of health system resources, including through the destruction of facility infrastructure, disruption of supply chains for medical supplies, and direct attacks or emigration of health workers (23–25). Chi et al. (23) also describe disruptions in the training of health workers, preferential emigration of skilled health workers, and overall reductions in the knowledge levels of health professionals. Fewer studies estimate the impact of conflict on structural or process quality quantitatively (26). Notably, a rigorous 2019 study by Akseer et al. (26) finds that equipment functionality reduced following moderate or severe intensity conflict, proxied by conflict-associated fatalities, between 2004 and 2010. Between 2011 and 2016, they do not find any impact of conflict intensity on equipment functionality. They also find no significant impact of conflict on drug availability, infrastructure adequacy and functionality, and service delivery interactions, including history, physical examinations, and client counseling. Akseer et al. (26) also document an increase in vaccine availability and health care professional knowledge in conflict-affected provinces. Hence, empirical estimations of the impact of conflict on the quality of care do not unambiguously align with findings described in the qualitative literature. There is a need for further evaluations of the implications of conflict for structural and process quality, across contexts, and an exploration of potential mechanisms for these effects empirically.

In this study, we focus on Kenya, given the availability of data on the quality of maternal health care through the Service Provision Assessment (SPA) that can be temporally and spatially linked to conflict events in the Armed Conflict Location and Events Data Project (ACLED) (27, 28). Kenya is the seventh most violent country in the ACLED, with over 3,500 politically violent events recorded between 1997 and 2013 (29). The multiple overlapping conflicts are attributable to a range of factors, including the politicization of ethnicity, inequities in land allocation, exploitation of local politics by Al-Shabaab, and the proliferation of small arms (30). Since 2010, there has been an increase in the intensity and frequency of violence, including terror attacks, conflicts between communities, and extra-judicial executions (31). Anecdotal accounts have linked these conflicts to subsequent disruptions in health and educational services (31). There are also significant disparities in maternal health outcomes in Kenya. Between 1990 and 2016, the MMR in Kenya fell from 315.7 to 257.6 deaths per 100,000 live births. However, maternal mortality increased in 25 of the 47 counties. Maternal mortality increased most substantially in Kirinyaga, by 4.9%, which is a county with a relatively high level of conflict incidence in Central Kenya (32). The coverage of ANC and SBA has remained low and inequitable in Kenya, with higher disparities in coverage for ANC (33, 34). However, studies examining the determinants of subnational disparities in maternal health care use in Kenya have not considered an independent role for conflict (35–37). There are also no published studies estimating the impact of conflict on the quality of health care in Kenya. Hence, in this study, we estimate the impact of conflict exposure on the quality of ANC in Kenya.

## Materials and Methods

### Data

The empirical analysis draws on data on the observed process and structural quality of ANC in the 2010 Kenya SPA, the locations of temporally and spatially matched conflict events in the ACLED, sociodemographic predictors of ANC use and quality in the SPA, and data on urbanicity of health facility locations from the Africover dataset.

The study sample was constructed from the 2010 Kenya SPA, which is a survey that collected nationally representative information from 695 health facilities, including hospitals, health centers, maternity and nursing homes, clinics, and stand-alone voluntary counseling and testing facilities offering maternal and child health care (27). The final sample covered ~11% of facilities in the country. Data collection commenced on 21st January 2010 and ended on 18th May 2010, which coincides with a year of increased conflict intensity and frequency in Kenya (31). In each facility, health service providers were sampled, if they were present on the day of the survey and provided one of the services being assessed. In facilities with fewer than eight health care providers, all the providers that were present were interviewed. In facilities with more than eight providers, not more than eight providers were interviewed. The survey includes responses from 3,051 providers.

Where many clients were present and eligible for observation, consultations were observed for up to 5 clients per provider and up to 15 clients per facility. To achieve the target number of observations, the total number of expected clients was divided by five to derive the nth interval for selecting the next client to be observed (27). A total of 1,409 ANC visits were observed. The Kenya SPA includes data on facility structural quality, including staffing levels, training and supervision of staff, availability of service delivery protocols, basic infrastructure (electricity, water, and client amenities), and systems for general facility management. Observations of ANC consultations assessed the process quality of care, including information shared between the client and provider, physical examinations, and treatment provided. Our analytical sample was restricted to the first ANC visit in a woman's pregnancy at the index facility, given that most recommended clinical content, for adequate process quality, occurs during this visit. This is consistent with recent studies that have examined the quality of ANC (38). Given that there was only one eligible ANC visit in May 2010, we focus the analysis on ANC visits that occurred between January and April 2010. Our analysis is robust to inclusion or exclusion of this datapoint. The survey also includes data on the geo-coordinates of the health facility, the date of the survey, as well as the sociodemographic characteristics of each provider and client.

We spatially linked the analytical sample with data from the ACLED, which documents the dates, actors, types of violence, locations, geo-coordinates, and fatalities of all reported political violence and protest events across Africa, South Asia, Southeast Asia, the Middle East, Central Asia and the Caucasus, and Southeastern and Eastern Europe and the Balkans. Finally, we derived information on the urbanicity of facility locations, by matching the geo-coordinates of each facility with urban areas as defined in the Africover dataset compiled by the Food and Agricultural Organization of the United Nations (39).

### Analysis

We estimated DID models of the impact of conflict on the quality of women's first ANC visits in Kenya, by comparing structural and process quality measures, during high- and low-conflict months, in health facilities at different levels of exposure to conflict.

We identified a total of 40 conflict events in Kenya within the ACLED that occurred between January and April 2010, overlapping with the timing of the analytical sample of ANC observations from the SPA. The ACLED events consist of violent events, demonstration events, and non-violent actions. Violent events in the ACLED are further classified as follows: battles, explosions/remote violence, and violence against civilians. Demonstration events are also classified into protests and riots, and non-violent actions in the ACLED are events related to strategic developments. The month of January is notable for recording 45% of all conflict events, 62% of all fatalities linked to conflict, 42% of violent conflict events, and 51% of fatalities linked to violent conflict (Table 1). Hence, regardless of the measure of conflict intensity considered, the month of January has a higher conflict intensity than other months. In our main models, we consider January to be a high-conflict month and compared January to the other months, which are considered low-conflict months. Our main models examine the impact of exposure to any conflict. We also examined the sensitivity of our findings to the type of conflict event, by limiting to violent and non-violent events, respectively.

**Table 1:** Temporal distribution of conflict events.

	**All conflict events**		**Violent conflict events**	
**Month**	***N***	**Fatalities**	***N***	**Fatalities**
January	18	28	9	18
February	11	5	6	5
March	7	10	3	10
April	4	2	3	2
Total	40	45	21	35

We determined facility exposure to conflict based on the distance from any conflict event within the ACLED sample to the facility location. We calculated the straight-line distance between each sampled facility to the location of all conflict attacks between January and April 2010. We classified a facility as exposed to a conflict event if any event was located within the catchment area of the facility. There is no consensus on the definition of the radius in which conflict may affect health care. Furthermore, the distances used to define catchment areas in health service research, within which physical access to care can be affected, in SSA, vary significantly (40). Hence, in our main models, we define the facility catchment area as a radius of 10,000 m. We examined the sensitivity of our findings to the definition of the catchment area radius, by varying this distance from 3,000 to 50,000 m.

We modeled the process and structural quality of ANC as a function of exposure to conflict and other determinants of high-quality maternal health care coverage, using DID models, specified as follows:


$$\begin{array}{l} Q_{ilt}=\beta_{0}+\beta_{1}HCM_{t}+\beta_{2}EF_{l}+\beta_{3}HCM_{t}\;*\;EF_{l}+\beta_{4}X_{ilt}+\varepsilon_{ilt}. \end{array}$$

Below, we describe the main empirical model in the order in which variables appear. All model variables are listed in Table 2.

**Table 2:** Model variables.

*Q_*ilt*_*	The score on an index of quality of first ANC visit *i* at facility *l* in month *t*; we used three index scores—process quality index score, equipment and infrastructure index score, and management and supervision index score
*HCM*_*t*_	A binary variable indicating if the ANC visit occurred in a high-conflict month *t*. *HCM_*t*_* takes the value 1 if the month was January, and 0 otherwise.
*EF*_*l*_	A binary variable indicating if the facility in which the ANC visit occurred was exposed to any conflict event, equal to 1 if the facility was within 10,000 m of a conflict event, and 0 otherwise.
*X*_*ilt*_	A vector of controls that are client-, provider-, and facility-level determinants of high-quality maternal health care use that were collected in the SPA, described below:
	- Educational attainment of the ANC client is a binary variable, coded as 1 if the individual had at least secondary education, and 0 otherwise.
	- Provider type is a binary variable, coded as 1 if the provider was a medical doctor, registered nurse, or registered midwife, and 0 otherwise.
	- Facility type is a binary variable, coded as 1 if the facility was a national-, provincial-, or district-level hospital (which correspond to secondary and tertiary health facilities), and 0 otherwise.
	- Urbanicity of facility location is a binary variable, coded as 1 if the facility was located in an urban area, and 0 otherwise.

*Q*_*ilt*_ is the score on an index of quality for a first ANC visit *i* at facility *l* in month *t*. We constructed three separate quality indexes—one process quality index and two structural quality indexes, of equipment and infrastructure and management and supervision, respectively.

We constructed an index of process quality drawing on 20 binary indicators of essential elements of the client–provider interaction during the first ANC visit, including history-taking, physical examination, laboratory investigations, treatment, and counseling, identified in the WHO recommended guidelines at the time (41). We calculated the percentage of items fulfilled in each visit, to develop a continuous index ranging from 0 to 100, whereby a higher score corresponded to greater adherence to the recommended clinical practices. A description of the components of the process quality index can be found in Table 3.

**Table 3:** Components of process quality index.

**A**	**History-taking**
1	Records maternal age
2	Asks about past pregnancy history
3	Asks about danger signs in current pregnancy
**B**	**Physical examination and laboratory tests**
4	Checks for pallor
5	Checks for edema
6	Examines breasts
7	Measures blood pressure
8	Measures client weight
9	Measures fundal height
10	Performs vaginal exam
11	Performs or refers for anemia test
12	Performs or refers for syphilis test
13	Performs or refers for HIV test
14	Performs or refers for urine tests
**D**	**Treatment and counseling**
15	Prescribed or gave iron or folic acid or both
16	Prescribed or gave tetanus toxoid injection
17	Discussed diet and nutrition during visit
18	Counseled on danger signs in pregnancy
19	Gave breastfeeding advice
20	Discussed delivery plans

We created indexes of structural quality of the ANC clinic and the facility using variables within the SPA dataset and consistent with recent studies of ANC quality (38). We then assigned to each ANC visit the structural quality score for each index on the day of that visit. The equipment and infrastructure index included 27 binary indicators of functional equipment, safety and sanitation items in the ANC clinic, and overall facility infrastructure. A separate index of management and supervision included 12 binary indicators of actions undertaken by district supervisors during facility visits and internal facility management processes, which have implications for the supervision of ANC services. We calculated the percentage of items that were fulfilled, to develop continuous indexes ranging from 0 to 100. A description of the components of the indexes can be found in Tables 4, 5.

**Table 4:** Components of equipment and infrastructure index.

**A**	**Functional equipment in ANC clinic**
1	Stethoscope
2	Sphygmomanometer
3	Exam light
4	Fetal stethoscope
5	Speculum
6	Functional scale
**B**	**Facility infrastructure**
7	Improved water source available year-round within 500 m
8	Ambulance with fuel or capacity to communicate with another facility
9	Central electricity or functional generator with fuel
10	Facility phone or short-wave radio available always
11	At least one functional client toilet observed (if observed an option)
12	Facility floor is swept clean, counters/tables/chairs are wiped and clean
13	Waiting room where clients are protected from sun and rain
14	System in place for maintenance of infrastructure
15	Sharps are adequately disposed of
16	Medical or contaminated waste is adequately disposed of
17	Specific equipment, such as speculum and forceps, wrapped in sterile paper, and sealed with tape
18	Medications are stored in a dry location, protected from water, sun, pests
19	Last medication supply within 4 weeks
20	All medications, vaccines, and contraceptives stored according to expiry date
21	Computer or stock ledger updated daily with medicine available
**C**	**Safety and sanitation items in ANC clinic**
22	All rooms have pourable water observed
23	All rooms have soap observed
24	All rooms have gloves observed
25	All rooms have sharps box observed
26	All rooms have surface disinfectant observed
27	All rooms have hand disinfectant observed

**Table 5:** Components of management and supervision index.

**A**	**Supervisor checks and discussion**
1	Last outside supervisory visit within 6 months
2	Supervisor checked registers
3	Supervisor discussed problems
4	Supervisor reviewed policy and administrative matters
5	Technical protocols are present to address service delivery issues
6	Supervisor held an official staff meeting
7	Supervisor observed service provision
**B**	**Facility management**
8	Last outside supervisory visit within 6 months
9	Personnel in ≥1 unit received Health Management Information System (HMIS) training in the past year
10	Facility includes HMIS unit
11	Management meeting once every 6 months
12	Staff community meeting within 6 months

The variable, *HCM*_*t*_, is a binary variable indicating if the ANC visit occurred in a high-conflict month and is equal to 1 if the month was January, and 0 otherwise. The variable, *EF*_*l*_, is a binary variable indicating if the facility in which the ANC visit occurred was exposed to any conflict event, equal to 1 if the facility was within 10,000 m of a conflict event, and 0 otherwise. The DID estimator can be conceptualized as the average difference between *HCM*_*t*_ = 1 and *HCM*_*t*_ = 0 in areas not within 10,000 m (*EF*_*l*_ = 0) subtracted from the average difference between HCM = 1 and HCM = 0 in areas within 10,000 m of a conflict even (*EF*_*l*_ = 1).

In our DID model, the parameter, β_3_, measures the impact of conflict on the quality of health care. We assume that in the absence of high intensity conflict, there would be no significant difference in the trends in the quality of ANC in conflict-exposed and -unexposed facilities. This is the corollary of the parallel trends' assumption given the absence of a distinct pre- and post-conflict period. We provide evidence in support of this assumption.

Lastly, the variable, *X*_*ilt*_, is a vector of controls that are client-, provider-, and facility-level determinants of high-quality maternal health care use that were collected in the SPA. We included a categorical variable for educational attainment of the ANC client, coded as 1 if the individual had at least secondary education, and 0 otherwise; a categorical variable for provider type, coded as 1 if the provider was a medical doctor, registered nurse, or registered midwife, and 0 otherwise; a categorical variable for facility type, coded as 1 if the facility was a national-, provincial-, or district-level hospital (which correspond to secondary and tertiary health facilities), and 0 otherwise; and a binary variable for urbanicity of facility location, coded as 1 if the facility was located in an urban area, and 0 otherwise. Our main model included client sampling weights and restricted to the 91% of observations for which no covariates were missing.

## Results

Of the 1,409 ANC visits observed, 553 were the first ANC visits in the client's pregnancy in the index facility and thus constitute the analytical sample. Of the 553 first ANC visits, 363 (66%) were made by women with primary-level education or less; 246 (44%) were attended by a doctor, registered nurse, or registered midwife; and 158 (29%) occurred in national, provincial, or district hospitals, whereas 103 (19%) occurred in facilities located in a rural area. A total of 87 visits (16%) occurred in January 2010. The number of clients that occurred within a facility exposed to any conflict varied with the catchment area radius, ranging from 32 visits (5.7%) at 3,000 m to 326 visits (59%) at 50,000 m. For the catchment area radius of 10,000 m, 79 visits (14.3%) occurred in health facilities exposed to conflict. These descriptive statistics are summarized in Tables 6, 7.

**Table 6:** Distribution of first ANC visits by month, client, provider, and facility characteristics.

**Variable**		**N (%)**
Client's education	Primary-level or less	363 (66)
	Secondary-level or higher	186 (33)
	Missing	4 (1)
Provider cadre	Doctor, registered nurse, or midwife	246 (44)
	Other health workers	307 (56)
Facility type	National, provincial, or district hospital	158 (29)
	Other health facilities	395 (71)
Area of residence	Rural	103 (19)
	Urban	450 (81)
Month	January	87 (16)
	February	187 (34)
	March	182 (33)
	April	98 (17)

**Table 7:** Distribution of first ANC visit by catchment area radius.

**Catchment area radius (m)**	**N (%)**
≤ 3,000	32 (5.7)
≤ 5,000	48 (8.7)
≤ 10,000	79 (14.3)
≤ 15,000	104 (18.8)
≤ 20,000	146 (26.4)
≤ 25,000	205 (37.1)
≤ 30,000	222 (40.1)
≤ 35,000	251 (45.3)
≤ 40,000	287 (51.9)
≤ 45,000	311 (56.2)
≤ 50,000	326 (59.0)

We examined the variation in process and structural quality scores within the analytical sample (Table 8). The process quality score was 70% on average. The most commonly received components of process quality were examination of blood pressure (94%), measurement of client weight (94%), measurement of fundal height (91%), and recording of maternal age (90%). The five least commonly received components were a vaginal examination (9%), counseling on breastfeeding (32%), nutrition counseling (53%), and discussion of danger signs in pregnancy (56%). The mean process quality score did not vary significantly by facility exposure to conflict, month, or characteristics of the client, provider, or facility (Table 9). There were also no significant differences in the percentage of ANC visits that received any of the 20 process quality components at the 5% level.

**Table 8:** Variation in ANC quality by facility exposure to conflict.

		**All visits**	**≤10 km**	**>10 km**	***p-*Value**
		**(%)**	**(%)**	**(%)**	
**Mean process quality score**		**70.3**	**69.4**	**70.4**	**0.766**
**A**	**History-taking**				
1	Records maternal age	90.3	90.7	90.2	0.916
2	Asks about past pregnancy history	63.2	61.9	63.4	0.859
3	Asks about danger signs in current pregnancy	86.0	82.1	86.7	0.461
**B**	**Routine examination and laboratory tests**				
4	Checks for pallor	88.6	88.9	88.5	0.936
5	Checks for edema	79.8	82.2	79.4	0.702
6	Examines breasts	59.0	36.3	62.8	0.013
7	Measures blood pressure	93.6	96.3	93.1	0.329
8	Measures client weight	93.9	97.6	93.3	0.164
9	Measures fundal height	90.8	95.3	90.0	0.080
10	Performs vaginal examination	8.6	5.7	9.0	0.456
11	Performs or refers for anemia test	77.3	85.5	75.9	0.108
12	Performs or refers for syphilis test	75.0	79.1	74.3	0.558
13	Performs or refers for HIV test	78.7	78.5	78.7	0.983
14	Performs or refers for urine tests	75.2	81.6	74.1	0.350
**D**	**Treatment and counseling**				
15	Prescribed or gave iron or folic acid or both	60.0	56.3	60.7	0.680
16	Prescribed or gave tetanus toxoid injection	79.2	81.0	78.9	0.710
17	Discussed diet and nutrition during visit	53.3	60.0	52.2	0.494
18	Counseled on danger signs in pregnancy	55.6	51.7	56.2	0.628
19	Gave breastfeeding advice	32.0	21.1	33.8	0.131
20	Discussed delivery plans	65.8	56.7	67.4	0.181
**Mean equipment and infrastructure score**		**70.2**	**69.5**	**74.6**	**0.117**
**A**	**Functional equipment in ANC clinic**				
1	Stethoscope	87.8	81.4	88.8	0.496
2	Sphygmomanometer	94.4	88.9	95.3	0.478
3	Exam light	36.3	45.7	34.7	0.322
4	Fetal stethoscope	87.8	100.0	85.8	0.000
5	Speculum	76.5	80.1	75.9	0.676
6	Functional scale	93.6	100.0	92.5	0.014
**B**	**Facility infrastructure**				
7	Improved water source available year-round within 500 m	84.3	74.6	86.0	0.269
8	Ambulance with fuel or capacity to communicate with another facility	94.6	95.6	94.5	0.811
9	Central electricity or functional generator with fuel	41.4	46.9	40.5	0.570
10	Facility phone or short-wave radio available always	94.6	95.6	94.5	0.811
11	At least one functional client toilet observed (if observed an option)	97.8	89.6	99.2	0.177
12	Facility floor is swept clean, counters/tables/chairs are wiped and clean	96.3	97.6	96.1	0.600
13	Waiting room where clients are protected from sun and rain	98.4	100.0	98.1	0.246
14	System in place for maintenance of infrastructure	40.5	57.2	37.7	0.090
15	Sharps are adequately disposed of	49.3	50.6	49.1	0.897
16	Medical or contaminated waste is adequately disposed of	18.8	4.5	21.2	0.002
17	Specific equipment, such as speculum and forceps, wrapped in sterile paper and sealed with tape	39.1	38.8	39.1	0.975
18	Medications are stored in a dry location, protected from water, sun, pests	64.3	76.9	62.2	0.184
19	Last medication supply within 4 weeks	44.6	72.1	40.2	0.005
20	All medications, vaccines, and contraceptives stored according to expiry date	48.0	51.8	47.4	0.703
21	Computer or stock ledger updated daily with medicine available	65.3	79.1	63.0	0.060
**C**	**Other items in ANC room**				
22	All rooms have pourable water observed	83.2	83.5	83.2	0.975
23	All rooms have soap observed	72.7	85.1	70.6	0.126
24	All rooms have gloves observed	88.5	100.0	86.6	0.000
25	All rooms have sharps box observed	98.1	100.0	97.8	0.084
26	All rooms have surface disinfectant observed	75.5	90.2	73.1	0.040
27	All rooms have hand disinfectant observed	30.9	40.9	29.2	0.284
**Mean management and supervision score**		**71.5**	**62.6**	**73.0**	**0.008**
**A**	**Supervisor checks/discussion**				
1	Last outside supervisory visit within 6 months	97.0	86.5	98.8	0.036
2	Supervisor checked registers	90.6	75.0	93.2	0.048
3	Supervisor discussed problems	93.3	83.6	94.9	0.090
4	Policy/administrative matters	68.9	53.4	71.5	0.125
5	Technical protocols or service delivery issues	83.8	66.9	86.6	0.059
6	Held an official staff meeting	56.4	39.7	59.2	0.081
7	Observed service provision	57.1	50.4	58.2	0.498
**B**	**Routine management**				
8	Last outside supervisory visit within 6 months	44.1	46.7	43.6	0.787
9	Personnel in ≥1 unit received HMIS training in the past year	23.9	10.5	26.3	0.014
10	Facility includes HMIS unit	94.8	100.0	93.9	0.001
11	Management meeting once every 6 months	84.4	100.0	81.9	0.000
12	Staff community meeting within 6 months	64.9	38.0	69.4	0.004

**Table 9:** Variation in ANC quality by month, client, provider, and facility characteristics.

**Variable**		**Process quality score**		**Equipment and infrastructure score**		**Management and supervision score**	
		**Mean**	***p-*value**	**Mean**	***p-*value**	**Mean**	***p-*value**
Client's education	Primary-level or less	69.2	0.130	68.4	0.005	72.2	0.252
	Secondary-level or higher	72.3		73.8		69.9	
Provider cadre	Doctor, registered nurse, or midwife	69.9	0.786	72.4	0.084	70.8	0.556
	Other health workers	70.6		68.4		72.1	
Facility type	National, provincial, or district hospital	69.8	0.752	77.0	<0.001	79.5	<0.001
	Other health facilities	70.5		67.5		68.3	
Area of residence	Rural	70.1	0.748	69.1	0.011	73.3	0.009
	Urban	71.1		75.0		63.8	
Month	January	75.6	0.194	72.0	0.149	70.2	0.374
	February	69.3		66.2		70.5	
	March	69.9		72.0		71.3	
	April	68.2		73.0		75.0	

The index of equipment and infrastructure was 70% on average. The majority of the facilities had all rooms equipped with boxes for sharps (98%), at least one functional client toilet (98%), an appropriate waiting room (98%), clean floor and surfaces (96%), and an ambulance or capacity to communicate with other facilities (95%). The least commonly observed components of the equipment and infrastructure index were adequate waste disposal (19%), an examination light in the ANC clinic (36%), appropriate storage of specific equipment, such as speculum and forceps (39%), central electricity or a functional generator with fuel (41%), and receiving the last medical supply within the past 4 weeks (45%).

Facilities located within 10,000 m of a conflict event were 14 percentage points more likely to have a fetal stethoscope, 7 percentage points more likely to have a functional scale, 16 percentage points less likely to adequately dispose of waste, and 32 percentage points more likely to have received a medication supply within the past 4 weeks, compared with facilities outside the catchment area radius. There were no other significant differences between unexposed and exposed facilities on components of the equipment and infrastructure score. The mean score for equipment and infrastructure was higher in facilities used by women with secondary education or above; in national, provincial, and district hospitals; and in urban areas. However, it did not significantly vary by provider cadre and month at the 5% level (Table 9).

The index of management and supervision was 71%. The most commonly observed components of management and supervision quality were receiving a supervisory visit within the last 6 months (97%), possessing a Health Management Information System (HMIS) unit (95%), discussing problems with a supervisor (93%), a supervisor checking the registers (91%), and a management meeting holding once every 6 months (84%). The least commonly observed components were a record of personnel receiving HMIS training in the past year (24%), having an external supervisory visit in the past 6 months (44%), a supervisor holding an official staff meeting (56%), a supervisor observing service provision (57%), and a staff community meeting having held within the past 6 months (65%).

On average, less facilities located within 10,000 m of a conflict event had personnel in more than one unit that had received training in HMIS in the past 12 months (by 16 percentage points), but more of these facilities had a HMIS unit (by 6 percentage points), a management meeting in the past 6 months (by 18 percentage points), and a staff community meeting within the past 6 months (by 31 percentage points). Overall, the mean management and supervision score in facilities within 10,000 m of a conflict event was 10 percentage points below the mean among facilities outside the catchment area radius. The mean management and supervision score was higher in national, provincial, and district hospitals, as well as in rural areas, and did not significantly vary by women's education, provider cadre, and month at the 5% level (Table 9).

In our main DID models, we show that ANC visits that occurred in facilities within 10,000 m of any conflict event in a high-conflict month received 18.0–21.1 percentage points fewer components of process quality on average. This effect was statistically significant at the 5% level, with or without adjustment for confounders. There was no significant difference in the mean equipment and infrastructure score, at the 5% level. However, facilities within 10,000 m of any conflict event in a high-conflict month had a mean management and supervision score that was 12.8–13.5 percentage points higher on average. This effect was also statistically significant at the 5% level, with adjustment for confounders. We summarize these results in Table 10.

**Table 10:** The impact of conflict exposure on ANC quality.

	**Process quality score**		**Equipment and infrastructure score**		**Management and supervision score**	
DID [*HCM*_*t*_ ^*^EF_*l*_]	−0.180	−0.211	0.000	0.004	0.135	0.128
95% CI	(−0.272, −0.088)	(−0.293, −0.130)	(−0.097, 0.096)	(−0.073, 0.082)	(−0.001, 0.272)	(0.020, 0.237)
*p-*Value	<0.001	<0.001	0.995	0.913	0.051	0.020
Control mean	0.691	0.640	0.691	0.623	0.736	0.751
R^2^	0.034	0.125	0.024	0.320	0.062	0.201
*N*	553	551	553	551	553	551
^*^Adjusts for covariates?	No	Yes	No	Yes	No	Yes

* *covariates in the fully adjusted models were client factors (age and education), provider factors (sex, provider cadre), and facility factors (facility type)*.

**Figure 1:**
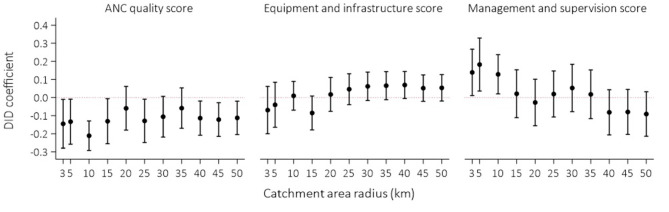
Variation in DID estimates with catchment area radius.

We examined the sensitivity of our findings to the exposure definition. First, we varied the catchment area radius from 3,000 to 50,000 m and re-estimated the DID models (Figure 1). We find that the point estimates of the impact of conflict exposure on process quality remained negatively signed and varied between −0.211 and −0.133 for catchment area radii below 10,000 m. However, the 95% confidence intervals overlapped with the null value between 20,000 and 35,000 m. Similar to the main models, there was no significant difference between exposed and unexposed facilities in the equipment and infrastructure score. At lower catchment area radii, where exposed facilities were closer to conflict, the point estimates of the impact were negatively signed. We find that the point estimates of the impact of conflict exposure on management and supervision were positive and statistically significant at the 5% level between 3,000 and 10,000 m and varied between 0.128 and 0.182 for catchment area radii below 10,000 m. As the catchment area radius increased, including facilities further away from the location of the conflict event, the point estimates become null and then negatively signed. The 95% confidence intervals overlap with the null value between 15,000 and 50,000 m. These findings are summarized in Table 11.

**Table 11:** Variation in DID estimates with catchment area radius.

**Radius (m)**	**ANC quality score**			**Equipment and infrastructure index**			**Management and supervision index**		
	**DID**	***p-*value**	**95% CI**	**DID**	***p-*value**	**95% CI**	**DID**	***p-*value**	**95% CI**
≤ 3,000	−0.145	0.035	(−0.280, −0.010)	−0.069	0.296	(−0.200, 0.061)	0.139	0.034	(0.011, 0.267)
≤ 5,000	−0.133	0.036	(−0.258, −0.009)	−0.040	0.527	(−0.165, 0.085)	0.182	0.015	(0.036, 0.329)
≤ 10,000	−0.211	<0.001	(−0.293, −0.130)	0.010	0.81	(−0.070, 0.089)	0.128	0.02	(0.020, 0.237)
≤ 15,000	−0.131	0.039	(−0.256, −0.006)	−0.085	0.075	(−0.179, 0.009)	0.021	0.756	(−0.111, 0.153)
≤ 20,000	−0.059	0.334	(−0.180, 0.061)	0.017	0.719	(−0.077, 0.111)	−0.027	0.677	(−0.156, 0.101)
≤ 25,000	−0.129	0.034	(−0.249, −0.009)	0.046	0.289	(−0.039, 0.132)	0.019	0.764	(−0.108, 0.147)
≤ 30,000	−0.106	0.066	(−0.219, 0.007)	0.062	0.122	(−0.017, 0.141)	0.053	0.428	(−0.078, 0.184)
≤ 35,000	−0.059	0.302	(−0.170, 0.053)	0.066	0.097	(−0.012, 0.144)	0.018	0.796	(−0.117, 0.152)
≤ 40,000	−0.114	0.018	(−0.208, −0.019)	0.069	0.068	(−0.005, 0.144)	−0.082	0.198	(−0.206, 0.043)
≤ 45,000	−0.122	0.01	(−0.215, −0.029)	0.052	0.164	(−0.021, 0.125)	−0.080	0.208	(−0.204, 0.045)
≤ 50,000	−0.112	0.017	(−0.204, −0.020)	0.054	0.150	(−0.019, 0.127)	−0.091	0.146	(−0.214, 0.032)

We then re-estimated the DID models, restricting to violent and non-violent conflicts, respectively. The ACLED defines a violent event as one that involves the exchange of armed force, or the use of armed force at close distance, between armed groups capable of inflicting harm upon the opposing side, including battles, explosions, and violence against civilians (42). Our findings do not qualitatively change following restriction to violent events or non-violent events only. The process quality score for visits in facilities within 10,000 m of a violent conflict event is 18.2 percentage points lower, whereas location within 10,000 m of a non-violent conflict event results in a score that is 22.1 percentage points lower on average, compared with facilities outside this radius. Both estimates are statistically significant at the 5% level. There is no statistically significant impact of conflict on the equipment and infrastructure score, irrespective of conflict type. The management and supervision score of facilities within 10,000 m of a violent conflict event is 20.4 percentage points above, whereas location within 10,000 m of a non-violent conflict event results in a score that is 9.3 percentage points higher on average, compared with facilities outside this radius. Both estimates are statistically significant at the 5% level. These results are summarized in Table 12.

**Table 12:** Variation in DID estimates with conflict type.

**Outcome**	**Conflict type**	**DID**	***p-*value**	**95% CI**	**R^**2**^**	**Control mean**	***N***
Process quality score	All	−0.211	<0.001	(−0.293, −0.130)	0.125	0.640	551
	Violent	−0.182	<0.001	(−0.271, −0.094)	0.123	0.643	551
	Non-violent	−0.221	<0.001	(−0.312, −0.130)	0.120	0.640	551
Equipment and infrastructure score	All	0.004	0.913	(−0.073, 0.082)	0.320	0.623	551
	Violent	0.038	0.307	(−0.035, 0.111)	0.307	0.628	551
	Non-violent	−0.001	0.853	(−0.104, 0.086)	0.328	0.625	551
Management and supervision score	All	0.128	0.020	(0.200, 0.237)	0.201	0.751	551
	Violent	0.204	0.001	(0.083, 0.325)	0.236	0.762	551
	Non-violent	0.093	0.113	(−0.022, 0.208)	0.190	0.746	551

We examined heterogeneity in the impact of conflict exposure on ANC quality by client, provider, and facility characteristics (Table 13). The DID estimates remained statistically significant at the 5% level for provider type, equipment and infrastructure index, management and supervision index, and for area of residence. We found that among women with secondary or more education, exposure to conflict had a more negative impact on process quality, with a score that was 23.1 percentage points lower on average relative to women with primary education or less. We also find that the positive impact of conflict exposure on the quality of management and supervision is driven by rural facilities. All other interaction terms are not statistically significant at the 5% level. We also examined effect modification *via* adequacy of facility equipment, infrastructure, management, and supervision on the impact of conflict exposure on the process quality of ANC. However, we did not find significant impacts at the 5% level (Table 14).

**Table 13:** Heterogeneity in conflict impact on ANC quality.

		**DID**			DID^*^ **covariate**					
**Outcome**	Covariate^*^	**Coef**.	***p-*value**	**95% CI**	**Coef**.	***p-*value**	**95% CI**	**Control mean**	**R^**2**^**	***N***
Process quality score	Secondary or more education	−0.085	0.105	(−0.188, 0.018)	−0.231	0.006	(−0.394, −0.067)	0.635	0.134	551
	Doctor, registered nurse, or midwife	−0.183	0.003	(−0.303, −0.062)	−0.04	0.665	(−0.220, 0.141)	0.636	0.126	551
	National, provincial, or district level hospital	−0.199	<0.001	(−0.302, −0.096)	−0.058	0.513	(−0.230, 0.115)	0.643	0.13	551
	Urban facility location	−0.198	0.009	(−0.345, −0.050)	−0.024	0.811	(−0.220, 0.173)	0.642	0.133	551
Equipment and infrastructure score	Secondary or more education	0.018	0.701	(−0.076, 0.112)	−0.014	0.807	(−0.127, 0.099)	0.621	0.325	551
	Doctor, registered nurse, or midwife	0.028	0.595	(−0.076, 0.131)	−0.041	0.569	(−0.182, 0.100)	0.626	0.328	551
	National, provincial, or district level hospital	0.020	0.682	(−0.077, 0.117)	−0.096	0.197	(−0.243, 0.050)	0.625	0.322	551
	Urban facility location	0.009	0.894	(−0.121, 0.138)	−0.011	0.916	(−0.219, 0.196)	0.625	0.325	551
Management and supervision score	Secondary or more education	0.085	0.209	(−0.048, 0.218)	0.111	0.151	(−0.041, 0.262)	0.747	0.215	551
	Doctor, registered nurse, or midwife	0.133	0.099	(−0.025, 0.291)	0.025	0.796	(−0.166, 0.216)	0.745	0.232	551
	National, provincial, or district level hospital	0.126	0.052	(−0.001, 0.253)	0.026	0.778	(−0.152, 0.203)	0.755	0.219	551
	Urban facility location	0.008	0.905	(−0.128, 0.145)	0.351	0.002	(0.135, 0.567)	0.751	0.243	551

* *The comparison groups: primary-level education or less, other health workers (outside doctors, registered nurses, or midwives), other health facilities (other than national, provincial, or district-level hospitals), and rural facility locations*.

**Table 14:** Interaction between process and structural ANC quality.

		**DID**			DID* **covariate**					
**Outcome**	**Covariate**	**Coef**.	***p*-value**	**95% CI**	**Coef**.	***p*-value**	**95% CI**	**Control mean**	**R^**2**^**	***N***
Process quality score	Equipment and infrastructure score	−0.629	0.260	(−1.727, 0.469)	0.534	0.460	(−0.886, 1.954)	0.542	0.143	551
	Management and supervision score	−0.363	0.101	(−0.797, 0.071)	0.206	0.498	(−0.390, 0.802)	0.679	0.127	551

Our DID models estimate the impact of conflict on ANC quality on the assumption that in the absence of conflict, there would be no significant difference in trends in the quality of ANC in conflict-exposed and -unexposed facilities. We provide evidence in support of this assumption by re-estimating the DID models, excluding the observations in the high-conflict month of January, and adjusting for confounders (Table 15). Hence, we compare ANC visits in facilities within 10,000 m of any conflict, and otherwise, in the months of February, March, and April. We assign the status of high-conflict month to February. We do not find significant differences in the process or structural quality of ANC care in facilities within 10,000 m of any conflict event, and otherwise, when we restrict to low-conflict months. This suggests that the effects we find in our main models are unlikely to reflect chance.

**Table 15:** Falsification test.

	**Process quality score**	**Equipment and infrastructure score**	**Management and supervision score**
DID [*HCM*_*t*_ *EF_*l*_]	0.040	0.510	0.071
95% CI	(−0.066, 0.145)	(−0.066, 0.168)	(−0.071, 0.213)
*p-*value	0.460	0.391	0.326
*N*	475	475	475

## Discussion

In this study, we estimate the impact of conflict exposure on ANC quality in Kenya, a country that has recorded subnational disparities in maternal health outcomes and an increase in multiple overlapping conflict events. We draw on observed indicators of the quality of patient–provider interactions, infrastructure and equipment, and management and supervision in 695 health facilities. Our study is one of the few that quantifies the impact of conflict on maternal health care quality in an African country. We find that when an initial ANC visit occurs in a facility within 10,000 m of any conflict event, in a high-conflict month, the client receives fewer recommended components of care in evidence-based clinical guidelines, but the facilities on average have higher management and supervision scores. We do not find any significant impact of conflict exposure on the mean equipment and infrastructure score for the ANC clinics and facilities. Our findings did not qualitatively vary in a statistically significant manner, at the 5 or 10% level, on limiting our analysis to violent or non-violent conflict events, varying the facility catchment area radius, or adjusting for confounders.

The determinants of gaps in access to high-quality ANC and other maternal health services are multifaceted, including poverty, lower educational levels, and physical distance from health facilities (35–37). In this study, we have demonstrated that conflict is also an important determinant of the quality of ANC that women receive in this context. Our study highlights the importance of designing and targeting maternal health policy based on the context-specific evidence on the mechanisms through which conflict affects health care. There is significant descriptive and quasi-experimental evidence from other contexts of the negative impact of conflict on facility infrastructure and equipment, including in the neighboring countries of Burundi and Uganda (23–25). However, we find that in Kenya, deterioration of equipment and infrastructure does not appear to be the main mechanism through which conflict has hitherto affected ANC quality. In Afghanistan, Akseer et al. (26) find negative impacts of conflict on equipment and infrastructure between 2004 and 2010 and no effect between 2011 and 2016. Our models demonstrate clear reductions in the process quality of ANC, which includes history-taking, physical examination, laboratory tests, and counseling, among facilities exposed to conflict in high-conflict months. This contrasts sharply with the study by Akseer et al. (26) who report no significant impacts of conflict on history, physical examination, and client counseling.

We did not find significant difference in the impact of violent and non-violent conflicts on the process quality of care. This aligns with the absence of a significant negative impact of conflict on equipment and infrastructure in the surveyed facilities and the absence of effect modification of the impact of conflict on process quality by the equipment and infrastructure index. Facilities in rural areas score 9.5 percentage points higher, on average, on the management and supervision score. We also find that when facilities are exposed to conflict, there is increased management and supervision support, particularly in rural areas. Since 1994, the Kenyan Government has systematically devolved health care decision-making. In 2010, a new constitution created a decentralized government, increasing district-level autonomy and financing for health care (43). Rural facilities appear to have benefitted from increased supervision support. It has been argued that management and supervision may play a role in increasing the resilience of service delivery to shocks, including conflict (44). However, in this study, management and supervision does not modify the negative impact of conflict on that quality of client–provider interactions in ANC. This finding aligns with studies that show that supervision has not meaningfully improved the quality of obstetric or sick childcare in African countries (45).

There are clear policy implications of our findings. Even in the absence of the destruction of equipment and infrastructure, following conflict, the quality of maternal health care may be negatively affected, as in Kenya. Hence, facilities with conflict events within their catchment area in Kenya should be targeted for support to improve client–provider interactions. While the impact of conflict does not vary with variation in management supervision in our sample, several critical components of supportive supervision were missing. For example, over 50% of facilities do not report an external supervisor visit in the past 6 months, and in 43% of cases, a supervisor does not observe actual service provision. These represent missed opportunities to detect and address gaps in service delivery interactions. Hence, it may be useful to explore if further improvements in supportive supervision increase the resilience of maternal health care delivery to conflict shocks. Future research may explore a potential role for other factors, including changes in provider motivation, in modulating the impact of conflict on process quality in Kenya (15).

Our study has limitations. We provide evidence in support of the assumption that there are no significant differences among exposed and unexposed facilities in the absence of conflict. However, in practice, this assumption cannot be definitively proven to hold. We demonstrate that our findings are robust to adjustment for confounding and variation in the definition of conflict and conflict exposure. The controls adjusted for in our models were constrained by data collected through the surveys. Hence, we were unable to adjust for some time-varying confounders of maternal health care utilization and quality, such as socioeconomic status, maternal age, parity, and decision-making autonomy. As we adjusted for client education, we consider this a reasonable proxy for some of the information that is unavailable, including socioeconomic status and decision-making autonomy. Given data limitations, we are also unable to explore the role of provider competence, intrinsic motivation, and external incentives in modifying the impact of conflict on process quality. Future studies would benefit from the use of primary data similar to the study by Akseer et al. Our study uses the latest SPA survey, conducted in 2010, highlighting the urgent need for investments in data on the quality of care. To explore the generalizability of our findings over time, future studies can explore if the impact of conflict on ANC quality has remained stable over time. Finally, our study was also underpowered to examine the impact of specific types of conflict events, such as protests or battles, on the quality of health care.

Despite these limitations, this study contributes to the emerging literature on the impact of conflict on the quality of maternal health care. Kenya is not classified by the World Bank Group as a fragile and conflict-affected state, due to a relatively strong institutional environment and the absence of a peace-keeping mission in recent years. Our findings buttress the fact that countries that do not meet this criterion may also experience negative impacts of conflict on health service delivery. Hence, the World Bank has acknowledged that the definition of a fragile and conflict-affected state may not take full account of contextual challenges, including fragilities in middle-income countries and the spatial dynamics of conflict (46). Given our findings, further research and policy that aims to improve the quality of care in facilities exposed to conflict in Kenya should focus on better understanding the determinants of the gaps in process quality, including provider motivation, competence, and incentives.

## Data Availability Statement

Publicly available datasets were analyzed in this study. This data can be found at: https://dhsprogram.com/data/available-datasets.cfm; https://acleddata.com/curated-data-files/; http://www.fao.org/geonetwork/srv/en/main.home (accessed February 16, 2021).

## Ethics Statement

This study involved a secondary analysis of anonymous data, collected as part of research protocols with ethical approvals obtained through the Demographic and Health Survey Program and the ACLED Project.

## Author Contributions

AC conceptualized the study. KW undertook the spatial and statistical analysis. AC, KW, and UE-M contributed to a review of the study methodology, interpretation of the results, drafting of the manuscript, and revisions of the intellectual content of the manuscript. All authors read and approved the final manuscript.

## Conflict of Interest

The authors declare that the research was conducted in the absence of any commercial or financial relationships that could be construed as a potential conflict of interest.
